# ROS-Induced DCTPP1 Upregulation Contributes to Cisplatin Resistance in Ovarian Cancer

**DOI:** 10.3389/fmolb.2022.838006

**Published:** 2022-02-09

**Authors:** Yu Wang, Peishi Chen, Xueping Chen, Daoyuan Gong, Yingsong Wu, Liping Huang, Yao Chen

**Affiliations:** ^1^ Obstetrics and Gynecology Center, Nanfang Hospital, Guangzhou, China; ^2^ School of Medical Laboratory and Biotechnology, Southern Medical University, Guangzhou, China; ^3^ Guangzhou Customs District technology center, Foshan, China

**Keywords:** ovarian cancer, DCTPP1, cisplatin, ROS, cisplatin resistance

## Abstract

Cisplatin resistance hinders the improvement of the prognosis of patients with ovarian cancer. Cisplatin induces cancer cell apoptosis by inducing reactive oxygen species (ROS). dCTP pyrophosphatase 1 (DCTPP1) is a newly discovered dNTP pyrophosphatase. This study aimed to identify the role of DCTPP1 in oxidative stress and cisplatin response of ovarian cancer. Our results indicates cisplatin-induced ROS generation was responsible for the upregulation of DCTPP1 in ovarian cancer cells, whereas DCTPP1 knockdown significantly enhanced the sensitivity of ovarian cancer cells to cisplatin, reflect in reactive oxygen species (ROS) generation, double-strand DNA breaks, and cell apoptosis. The expression of redox-related genes and the activation of the PI3/Akt signaling pathway were also inhibited by DCTPP1 knockdown. Our data proposes that the development of therapeutic approaches targeting DCTPP1 may be useful in the treatment of ovarian cancer.

## Introduction

The lack of specific symptoms and early diagnostic markers have made ovarian cancer the most deadly gynecological cancer among women in the world. The current standard treatment for ovarian cancer is surgery accompanied by platinum-based chemotherapy (Brett et al., 2017; Webb and Jordan, 2017). In the past few decades, because of chemotherapy resistance, there has been only a very mild improvement in the survival rate of ovarian cancer patients. Thus, the mechanism of cisplatin resistance needs to be further elucidated to improve the outcome of patients with ovarian cancer (Vlahos et al., 2010; Glubb et al., 2021).

According to reports, cisplatin can induce the ROS-dependent apoptosis in ovarian cancer. ROS is a double-edged sword for tumor development (Fruehauf and Meyskens, 2007; Marullo et al., 2013; Srinivas et al., 2019). High-speed proliferation, resistance to apoptosis, increased migration and invasiveness, several of these features are known to be mediated by reactive oxygen species (ROS). However, oxidative DNA damage caused by ROS might lead to cell senescence or cell death (Han et al., 2019; Kleih et al., 2019; Wang et al., 2020).

Hence, tumour cells must deal with the oxidative DNA damage of ROS and maintain a suitable high level of ROS at the same time. One way for tumor cells to achieve this purpose is to balance the intracellular ROS levels by increasing the expression of redox protective proteins (Srinivas et al., 2019; Azzouz et al., 2021). Such an adaption would serve to uncouple the tumor-promoting effects of ROS from their tumor-suppressive consequences, and also contribute to the resistance to cytotoxicity of platimuns (Girish et al., 2013; Preya et al., 2017; Li et al., 2019; Rai and Sobol, 2019). The inhibition of redox-protective proteins could significantly sensitize such cells to platinums despite their not having a direct role in cell survival (Girish et al., 2013; Azzouz et al., 2021). As such, identifying the members in this class of proteins is likely to acquisit therapeutic targets with clinically valuable and/or prognostic markers for platinmuns therapy (Rai et al., 2011; Li et al., 2019; Rai and Sobol, 2019).

Detoxification of oxidative damage to DNA precursors is an important downstream mediator of ROS-induced cellular responses (Pecsi et al., 2011; Rai et al., 2011; Lu et al., 2018). Nucleotide triphosphate pyrophosphatase (NTP-PPase) is believed to hydrolyze abnormal nucleotides in cancer cells to maintain genetic stability, so that tumor cells can proliferate and survive under oxidative stress (Pecsi et al., 2011; Rai, 2012; Lu et al., 2018; Martinez-Arribas et al., 2020). dCTP pyrophosphatase 1 (DCTPP1) is a newly discovered dCTP pyrophosphatase with a MAGZ structure. Compared with the NTP-PPase which has been found to hydrolyze dGTP and dUTP and their derivatives, the substrate of DCTPP1 is dCTP, 5-methyl Base-dCTP and 5-halo-dCTPs etc. DCTTP1 exhibits nucleic accumulation in multiple carcinomas (Requena et al., 2014; Requena et al., 2016). Researches have shown that DCTPP1 promotes proliferation of breast cancer via DNA repair signaling pathway (Requena et al., 2016; Xia et al., 2016; Niu et al., 2021). Recently, the regulatory function of DCTPP1 in drug response has gained attraction. DCTPP1 attenuates the sensitivity of human gastric cancer cells to 5-fluorouracil by up-regulating MDR1 expression epigenetically and DCTPP1 is involved in the cellular response to decitabine (Xia et al., 2016; Niu et al., 2021). However, the role of DCTPP1 in response to cisplatin in ovarian cancer cells remains largely unknown.

In the present study, we investigated the role of DCTPP1 in regulating oxidative stress and homeostasis in ovarian caner cells under cisplatin treatment. We show that DCTPP1 amplified antioxidant capacity of OC cells, reflected in reducing oxidative stress–induced DNA damage and cisplatin sensitivity. Our results have provided new insights into potential therapeutic strategies to overcome cisplatin resistance in ovarian cancer.

## Materials and Methods

### DCTPP1 Knockdown in Ovarian Cancer Cells

Human ovarian cancer cell lines SKOV3 and OCAVAR8 were purchased from the cell bank of the Chinese Academy of Sciences (Shanghai, China) and cultured in DMEM culture solution containing 12% fetal bovine serum, respectively, and placed in an incubator at 5% CO2, 37°C. Lentivirus contain DCTPP1 shRNA (target:GCCCTTCAAGAGGAGCTTA) and negative controls (5′-GTT​CTC​CGA​ACG​TGT​CAC​GT-3′) were purchased from Shanghai Gima Pharmaceutical Co., Ltd. SKOV3 and OCAVAR8 cells in logarithmic growth phase were planted in 24-well plates with a planting density of 5 × 10^4^/well. When the cell growth density reached 50%, infection was performed for 24 h according to the instructions and then transected cells are selected by puromycin to establish DCTPP1 knockdown cell. The cells of each group were expanded cultured for follow-up work.

### qRT-PCR Analysis

Ovarian cancer cells were lysed by Trizol method, then total RNA was extracted and synthesized into cDNA.qPCR assay was performed follow the instruction manual of SYBR premix Ex Taq Kit. qPCR reaction program was set to two steps: (a) pre-incubation at 95°C for 30 s; (b) 95°C for 5 s and 55 °C for 30 s, 72 °C for 34 s for 40 cycles. Samples were assayed in triplicate using the ABI Prism 7500 detection system (Applied Biosystems, Foster City, CA, United States). The relative quantization value was then calculated by subtracting the average CT from the corresponding average CT for 18SRNA.

### Western Blot Analysis

Cells were harvested and lysed in ice-cold lysis buffer (10 mM Hepes, pH 7.9; 150 mM NaCl; 1 mM EDTA); The protein concentration of cell Lysate was determined by BCA protein assay kit. Samples were separated by 10% or 12% sodium dodecyl sulfate polyacrylamide gel electrophoresis (SDS-PAGE) and transferred to polyvinylidene difluoride (PVDF) membranes (Millipore,United States), which was then blocked in 5% BSA blocking buffer at 4°C overnight. All antibodies (dilution, 1:1,000; Santa Cruz Biotechnology, Santa Cruz, CA, United States) were incubated on shaking bed overnight at 4°C respectively. Secondary antibody was incubated at room temperature for 30 min.Densitometric quantification of protein bands were visualized using ECL-Plus detection reagents (Santa Cruz Biotechnology, Santa Cruz, CA, United States) and performed with GAPDH as an internal control using ImageJ.

### Luciferase Reporter Assay

The genomic DNA was routinely extracted according to the instructions of the genome extraction kit. Two different promoter sequences of DCTPP1 (P1: 2000 -0 bp upstream of DCTPP1 coding sequence; P2:674-0 bp upstream of DCTPP1 coding sequence) were cloned and inserted into the luciferase vector. The luciferase reporter plasmids named luc-2000 and luc-674 respectively, were transfected into cells with empty vector as a control. The Dual luciferase reporter assay kit (promega, Madison, Wisconsin, United States) was used to detected the luciferase signal.

### Cell Viability Assay *in vitro* (CCK-8)

Cells (1 × 10^3^/100 μL) were seeded in 96-well plates (Corning, New York, United States) and cell viability was analyzed by using CCK-8 kit (Dojindo Laboratories, Kumamoto, Japan) according to the manufacturer’s instructions. The absorbance of 450 nm was measured on a microplate reader (BioRad, CA, United States) and the cell growth curves were calculated.

### Flow Cytometry for Apoptosis Analysis

Cells (1 × 10^5^) were Collected for cell apoptosis assay using propidium iodide (PI, Sigma, Shanghai, China) and apoptosis assay using FITC-Annexin V Apoptosis Detection Kit (Biolegend, San Diego, CA, United States) according to the manufacturer’s instructions. Stained Cells (10,000 events for each sample) were acquired by FACS (BD Pharmingen, San Diego, CA, United States) and the results were analyzed with MLFT32 software.

### Rreactive Oxygen Species Level Detection

ROS levels were detected using dihydroethidium (DHE) (Sigma-Aldrich, CA, United States). Cells were harvested and incubated with DHE (10 µ M) for 10 min at 37°C in the dark. Relative red fluorescence was measured using a flow cytometer (BD Pharmingen,CA, United States).

### 3D Growth Model Build by Collagen Gel Droplet Culture Technology (CD-DST)

Briefly, type I collagen (Cellmatrix Type CD; Nitta Gelatin, Inc., Osaka, Japan), 10× F-12 medium, and reconstitution buffer were mixed together at a ratio of 8:1:1. The prepared tumor cells and collagen solution are inoculated at a volume ratio of 10:1, so that the final density of cells in the collagen droplets is 2 × 10^5^–5 × 10^5^ pieces/mL. The collagen-cell mixture (30 μL/drop) was transferred to the 6-well multiplate and cultured at 37°C in a CO_2_ incubator. After culture for 16 days, Each collagen droplet was stained with neutral red, fixed with 10% neutral formalin buffer, washed with water, and quantified by culture cell analysis system. Image analysis at 540 nm would quantify the number of DCTPP1 knockdown cells and control cells.

### Immunohistochemistry

Ovarian cancer specimens are surgical resection specimens, the specimens are fixed with 10% neutral formaldehyde after removal from the body. Immunohistochemistry was done using 8 μm serial sections placed onto glass slides using a single-staining procedure.

The protocols used with DCTPP1 and Ki67 antibody (dilution, 1:50; Santa Cruz Biotechnology, Santa Cruz, CA, United States) are carried out according to the instructions of Immunohistochemistry kit, and negative and positive controls were seted. Double-blind reading method was used to judge the staining results: two experienced doctors read the pictures separately, 10 representative fields were randomly observed in each slice, and comprehensive analysis and scoring was performed according to the respective staining degree and number of stained cells.

### Comet Assay

Alkaline unwinding and electrophoresis conditions was carried out according to instructions for the comet assay kit (Phgene, KM,China). A minimum of 100 individual cells per sample were scored in duplicate from three independent experiments. According to the DNA tail lengths, cell damage is divided into three levels: level 1, none (no damage); level 2, medium (moderate damage); level 3, long (severe damage);

### Statistical Analysis

Graphpad prism 5.0 and Excel 2016 was used to generate the graphs and process the data. The significance was analyzed with the unpaired t-test and one-way ANOVA, *p* values < 0.05 were considered significant. Data were expressed as the mean ± SD from at least three independent experiments.

## Result

### Overexpression of DCTPP1 is a Poor Prognostic Marker in Ovarian Cancer

**FIGURE 1:**
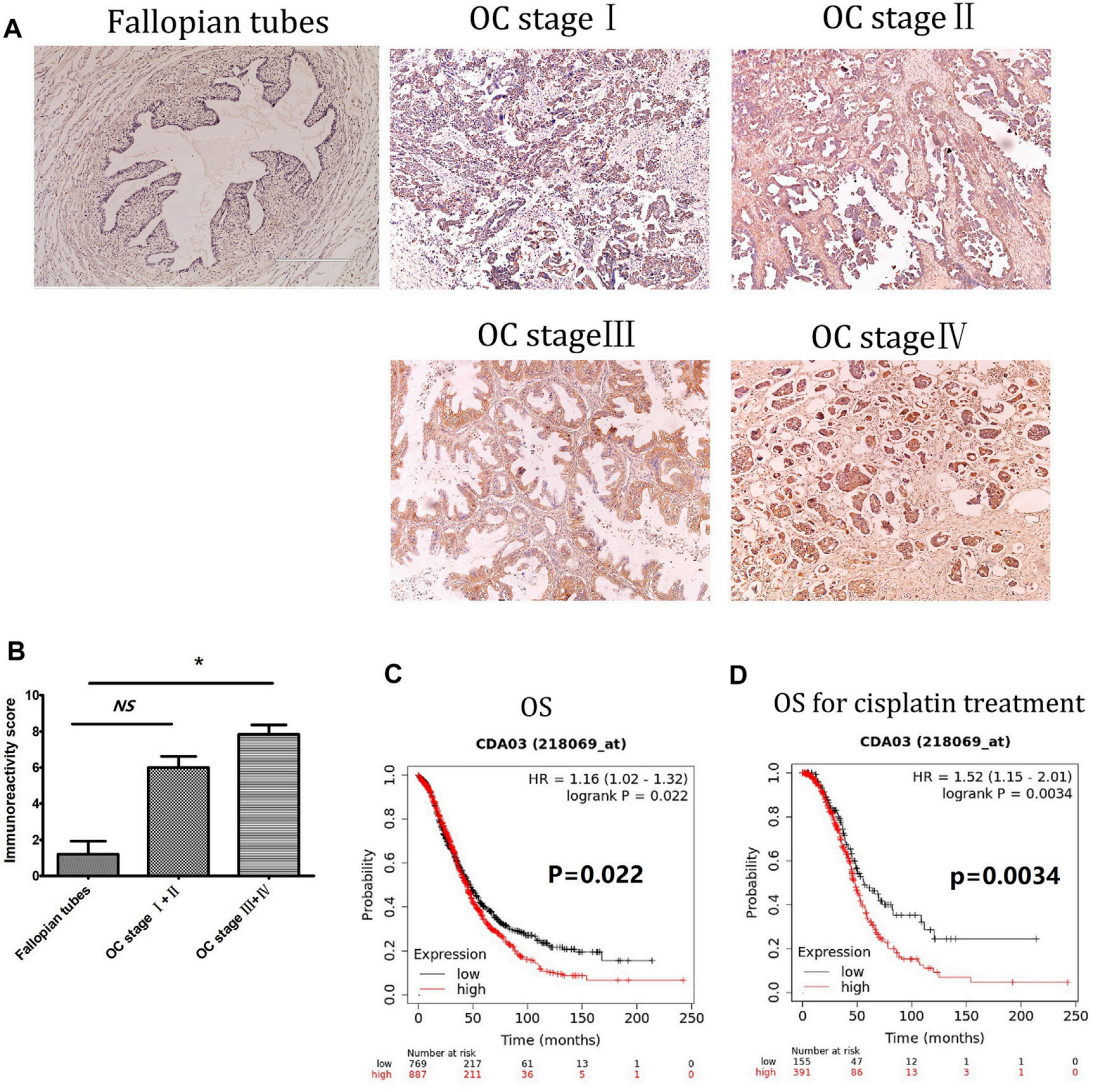
Overexpression of DCTPP1 is a poor prognostic marker in ovarian cancer. **(A)** Immunohistochemistry staining of DCTPP1 on human Fallopian tubes and ovarian cancer tissues. **(B)** Scoring of DCTPP1 staining in human ovarian cancer tissues with different pathological grade (Fallopian tubes, stage Ⅰ + Ⅱ, stage Ⅲ + Ⅳ).*,*p* < 0.05; ns, no significance. **(C)** Kaplan-Meier analysis of overall survival (OS) in ovarian cancer patients based on DCTPP1 expression level. **(D)** Kaplan-Meier analysis of overall survival (OS) in ovarian cancer patients with cisplatin treatment based on DCTPP1 expression level.

Immunohistochemistry was used to measure DCTPP1 protein expression, represented by immunoreactivity score (IRS) in OC tissues and adjacent non-cancerous fallopian tubes tissues. As shown in Figure 1A, Expression of DCTPP1 was weakly or almost no stained in non-cancerous fallopian tubes tissues, positive signals of DCTPP1 was detectable mainly in the nucleus and cytoplasm of cancerous tissues. Statistical analyses indicated that DCTPP1 expressions in OC tissues of Ⅲ and Ⅳ stage are significantly higher than that inadjacent non-cancerous fallopian tubes tissues with *p* < 0.05 (Figure 1B).

Correlation between DCTPP1 expression and clinicopathological parameters was further analyzed, the expression of DCTPP1 was higher in older patients (>56 years old), no evident correlations were observed between DCTPP1 expression with the FIGO stage, lymph node and omentum metastasis, and CA125 (Table 1). The correlation between DCTPP1 expression and disease prognosis was analysis using the Kaplan–Meier assay. As shown in Figure 1C, 887 patients in the DCTPP1-high group exhibited a poorer prognosis when compared with 768 patients in the DCTPP1-low group (*p* = 0.022, Figure 1C). These results indicate that DCTPP1 is strongly associated with ovarian cancer prognosis and might play some role in ovarian cancer progression. And under the condition of cisplatin treatment, there was significant difference in the overall survival (*p* = 0.0034) between the patients in DCTPP1-high group and the patients in DCTPP1-low group. This results suggested that patients with high expression of DCTPP1 had a poor response to cisplatin treatment (Figure 1D).

**TABLE1:** Correlation between *DCTPP1* expression and clinicopathological characteristics.

Clinicopathological features		*DCTPP1* expression		
		Low expression	High expression	*p* Value
Age (years)	<56	44	6	0.0060*
	≥56	26	15	
FIGO staging	I + II	21	5	0.75
	III + IV	49	14	
CA125(U/ml)	<600	41	13	0.47
	≥600	24	5	
Lymph node metastasis	positive	17	1	0.06
	nagetive	52	18	
Transcoelomic Metastasis	positive	43	13	0.58
	nagetive	27	6	

^*^ P < 0.05,compared to the low expression group.

### DCTPP1 Expression Could be Induced by Cisplatin Through ROS Generation

**FIGURE 2:**
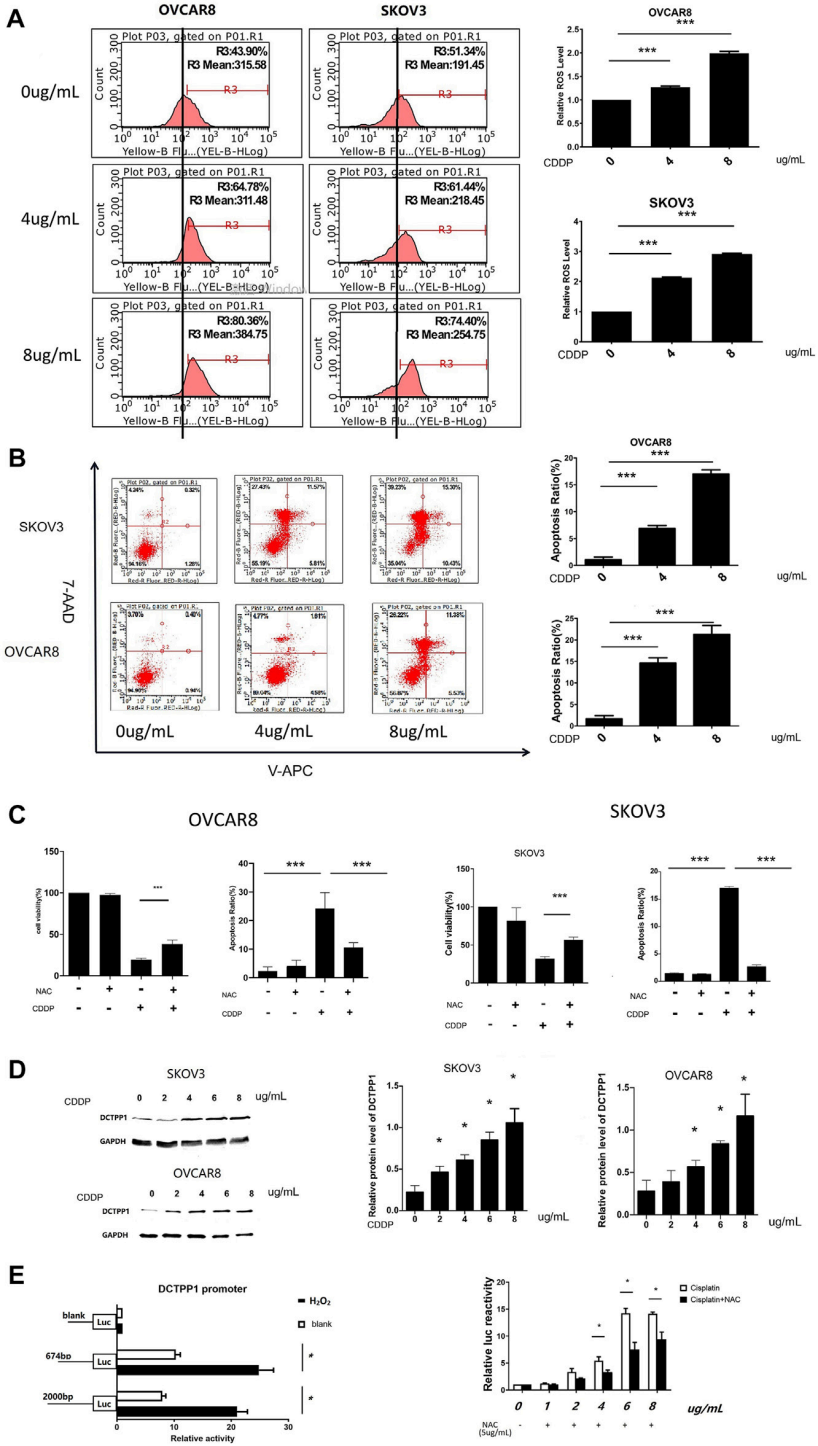
DCTPP1 expression could be induced by cisplatin through ROS generation. **(A)** The level of reactive oxygen species (ROS) was detected using DHE in OVCAR8 and SKOV3 cells treated with different concertration cisplatin (0, 4, and 8 μg/ml); ***,*p* < 0.001; B. Cell apoptosis ratio was analyzed with flow cytometry in OVCAR8 and SKOV3 cells treated with different concertration cisplatin (0,4,8 μg/ml); ***,*p* < 0.001; **(C)** Statistical analysis of cell viability and apoptosis ratio exposed to cisplatin (4 μg/ml) with or without NAC(5 μg/ml). *p < 0.05; **,*p* < 0.01; ***,*p* < 0.001; **(D)** Western blot analysis of SKOV3 and OVCAR8 cells treated with cisplatin (0,2,4,6,8 μg/ml). *p < 0.05; **,*p* < 0.01; ***,*p* < 0.001; **(E)** luciferase reporter assay analysis of DCTPP1 promoter luciferase reporters in SKOV3 cells treated with different concertration cisplatin (1, 2, 4, 6, and 8 μg/ml) and NAC(5 μg/ml),*p < 0.05.

In order to determine the role of ROS in cisplatin-induced cytotoxicity, we tested the level of ROS generation in the two ovarian cancer cells after cisplatin treatment. The results showed that cisplatin increased ROS levels and correspondingly increased apoptosis ratio in SKOV3 and OVCAR8 cells (Figures 2A,B), while cisplatin-induced apoptosis decreased when N-acetyl-l-cysteine (NAC) was added (Figure 2C). These results suggest cisplatin-induced generation of reactive oxygen species (ROS) has been correlated to its cytotoxic effects, which makes specific ROS scavengers being great significance to cisplatin resistance.

As shown in Figure 2D, induction effect of cisplatin on DCTPP1 was observed in both SKOV3 and OVCAR8 cells at the protein level after cisplatin treatment (*p* < 0.05). These results suggest that DCTPP1 might be a response factor for cisplatin. And then, we investigated our hypothesis that DCTPP1 expression could be induced by cisplatin through ROS generation. The dual luciferase reporter gene system is used to efficiently detect the transcriptional activation of DCTPP1. As shown in Figure 2E, the DCTPP1 promoter can be activated by the ROS inducer H_2_O_2_ (500uM), suggesting that DCTPP1 is a response factor of oxidative stress. At the same time, the activation of the DCTPP1 promoter by cisplatin can be reversed by N-acetyl-l-cysteine (NAC), the oxidative stress inhibitor (*p* < 0.05, Figure 2E). These results show that intracellular ROS accumulation induced by cisplatin up-regulated the transcription of DCTPP1. In summary, DCTPP1 expression could be induced by cisplatin through ROS generation.

### Knockdown of DCTPP1 Inhibits Tumor Growth *via* Regulating the Akt Pathway

**FIGURE 3:**
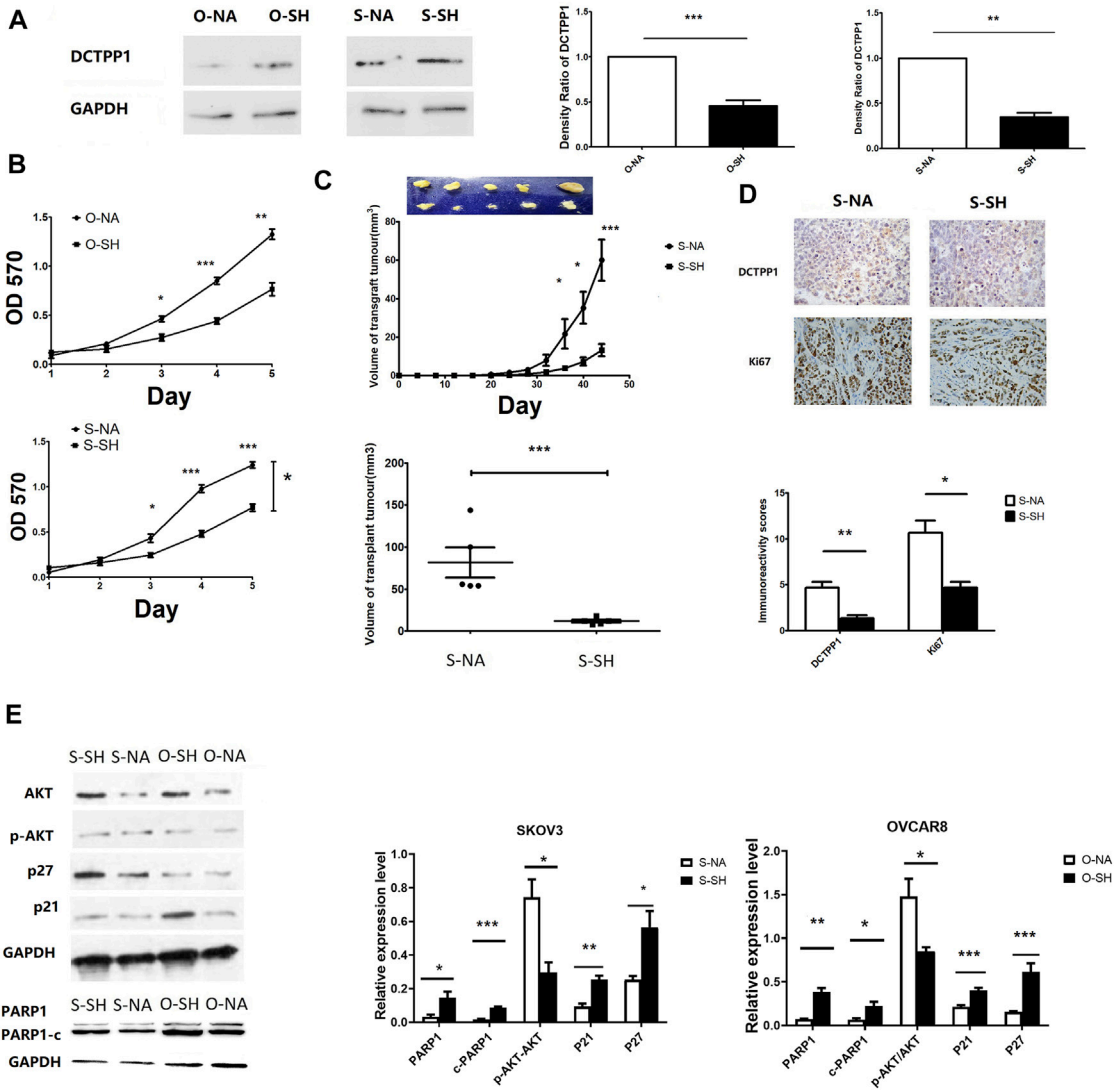
Knockdown of DCTPP1 inhibits tumor growth via regulating the Akt pathway. **(A)** Western blot analysis and densitometric quantification of DCTPP1 in SKOV3 and OVCAR8 cells treated with shRNA against DCTPP1. **,*p* < 0.01; ***,*p* < 0.001; **(B)** Growth curve of SKOV3 and OVCAR8 cells with the indicated shRNA transpected.*p < 0.05; **,*p* < 0.01; ***,*p* < 0.001; **(C)** Up:Mean tumor growth curves for xenografts from SKOV3 cells transfected with shRNA and the control respectively. *p < 0.05,***,*p* < 0.001; compared to S-NA; Down: Mean tumor volume of xenografts from SKOV3 cells transfected with shRNA and the control respectively at 6 weeks***,*p* < 0.001; **(D)**. DCTPP1 and Ki67 immunohistochemistry in SKOV3 cells transfected with shRNA and the control respectively; *p < 0.05; **,*p* < 0.01; **(E)** Western blot analysis and densitometric quantification of P21,P27,PARP1,c-PARP1,Akt,p-Akt in ovarian cancer cells transfected with shRNA and the control respectively. Data are presented as mean ± SEM.*p < 0.05; **,*p* < 0.01; ***,*p* < 0.001.

Figure 3A shows cell lines with stable DCTPP1 knockdown were constructed, and the knockdown efficiency was assessed by Western blot respectively (*p* < 0.05). The OVCAR8 and SKOV3 cell lines with stable knockdown of endogenous DCTPP1 by short hairpin RNA (shRNA) were named as S-SH and O-SH respectively, the control cell lines were named S-NA and O-NA respectively. These four cell lines were selected for use in subsequent experiments.

CCK-8 assay were conducted to determine the cell growth. As shown in Figure 3B, the growth curves indicated that the growth rate was significantly lower in DCTPP1 knockdown cells than the control cells. A xenograft tumor mouse model was established to identify the impact of DCTPP1 on tumour growth *in vivo*. As shown in Figure 3C, loss of DCTPP1 expression resulted in a significant delay in tumor growth, where the difference in tumor volume was statistically significant after 6 weeks (*p* < 0.05). Ki67, a marker of tumour proliferation, was also observed to be decreased in DCTPP1 knockdown groups by immunohistochemistry (Figure 3D). As mounting evidence indicates that PIK3/Akt pathway always plays a crucial role in tumour growth, DCTPP1 deletion in both SKOV3 and OVCAR8 cells resulted in an elevated level of p21 and, at the same time, a decreased level of pAkt/Akt (Figure 3E). These results might predict the DCTPP1 knockdown would sensitize the sensitivity of cisplatin of ovarian cancer cells. Correspondingly, we have also observed an increase in the expression of apoptosis-related proteins PARP1 and c-PARP1, which suggested that the knockdown of DCTPP1 may stimulate cell apoptosis by down-regulating the activation of the Akt pathway.

### DCTPP1 Knockdown Induces ROS Overproduction in Ovarian Cancer Cells Under Cisplatin Treatment

**FIGURE 4:**
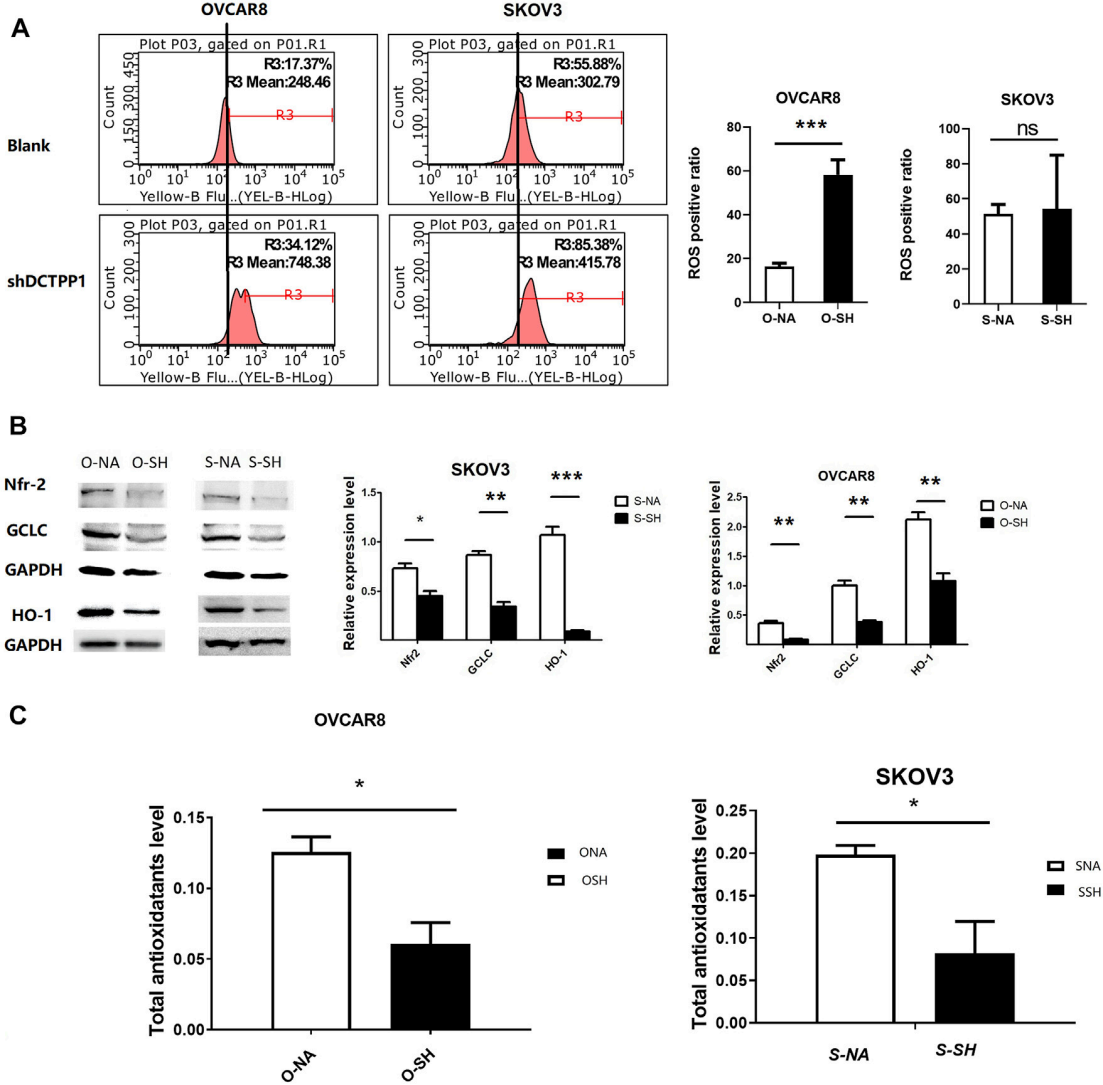
DCTPP1 knockdown induces ROS overproduction in ovarian cancer cells. **(A)** The level of reactive oxygen species (ROS) was detected using DHE in control or DCTPP1 knockdown cells.***,*p* < 0.001; ns, no significance. **(B)** Western blot analysis and densitometric quantification of Nrf2, GCLC and HO-1in ovarian cancer cells transfected with shRNA and the control respectively. Data are presented as mean ± SEM. *p < 0.05; **,*p* < 0.01; ***,*p* < 0.001; **(C)** Total antioxidotatants level of ovarian cancer cells transfected with shRNA and the control respectively. Data are presented as mean ± SEM.∗ *p* < 0.05.

Cisplatin is a well-known inducer of ROS and our previous researches have demonstrated that the cytotoxic effect of cisplatin was related to the increasing intracellular ROS level. We hypothesized that DCTPP1 promotes resistance of ovarian cancer cells to cisplatin-induced intracellular ROS production. To reveal the effect of DCTPP1 knockdown on intracellular ROS generation, we detected the total ROS levels in ovarian cancer cells using DHE staining. Although the effect of DCTPP1 knockdown on the level of intracellular ROS is not obvious, it has certain statistical significance (Figure 4A, *p* < 0.05) in OVACAR8 cells. It is worth noting that we have observed a significant downregulation of Nrf2 after DCTPP1 knockdown (Figure 4B, *p* < 0.05). More importantly, DCTPP1 knockdown also led to a decrease in glutamate-cysteine ligase, catalytic subunit (GCLC) and heme oxygenase 1 (HO-1), two of the major antioxidants located downstream of Nrf2 to suppress oxidative stress (Figure 4B, *p* < 0.05). These results suggest that overexpression of DCTPP1 in ovarian caner might activate the Nrf2-dependent antioxidative response in ovarian cancer cells, thus avoiding the oxidative stress and maintaining survival. Correspondingly, we observed changes in the total antioxidant capacity in the cells. DCTPP1 knockdown reduced the total antioxidant capacity (Figure 4C, *p* < 0.05), which further proved that DCTPP1 can participate in the redox homeostasis of the cell by regulating the Nrf-2-dependent antioxidant response.

### DCTPP1 Knockdown Increases Cisplatin-Sencitivity in Cisplatin-Sensitive Ovarian Cancer Cells

**FIGURE 5:**
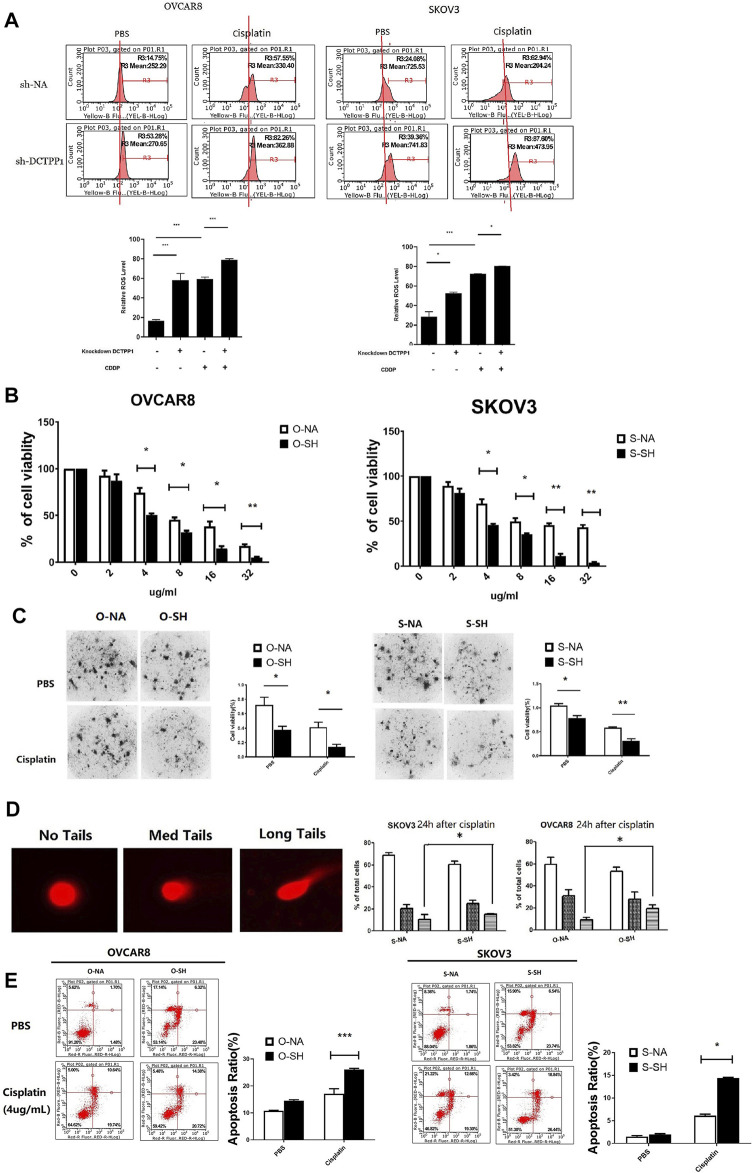
DCTPP1 knockdown increases cisplatin-sencitivity in cisplatin-sensitive ovarian cancer cells. **(A)** The level of reactive oxygen species (ROS) was detected using DHE in control or DCTPP1 knockdown cells treated or without treated with cipalin (4 μg/ml).*p < 0.05; ***, *p* < 0.001.**(B)** DCTPP1 knockdown inhibit cell viability both in SKOV3 and OVCAR8 cells treated with cisplatin of different concentration (0, 2, 4, 8, 16, and 32 μg/ml). *p < 0.05; **,*p* < 0.01; **(C)** Clone-formation of ovarian cancer cells infected with lentiviral DCTPP1 shRNA and a control shRNA, was measured by the CD-DST assay. *, *p* < 0.05; **, *p* < 0.01. **(D)** Alkaline comet assay data is shown the indicated samples. Representative single cell images used for scoring within the three categories are shown next to the quantitation. *p < 0.05. **(E)** Apoptosis was analyzed by flow cytometry. Representative plots showing the redistribution of phosphatidylserine (annexin V staining) in the presence of propidium iodide (PI). Statistical analysis of apoptosis of cells exposed to PBS and cisplatin, respectively. *p < 0.05; ***, *p* < 0.001.

To determine whether there was a causal link between cisplatin-induced upregulation of DCTPP1 and cisplatin-sensitivity, we observed the intracellar ROS in DCTPP1 knockdown cells under cisplatin treatment compare to the control. Compared to their parental cell lines, significantly higher intracellular ROS concentrations were detected after DCTPP1 knockdown, which indicated that DCTPP1 knockdown significantly increases intracellular ROS levels in ovarian caner cells cotreated with cisplatin (*p* < 0.05, Figure 5A). These results verified our hypothesis that the high expression of DCTPP1 may rebalance the excessive accumulation of ROS induced by cisplatin treatment. DCTPP1 knockdown might cause more oxidative stress in ovarian cancer cells under cisplatin treatment. The viability of SKOV3 and OVCAR8 cells exposed to cisplatin with or without DCTPP1 knockdown were assessed by CCK-8 assay and CD-DST assay. There is significant difference in cell viability between DCTPP1 knockdown group and the control group both in CCK-8 assay (*p* < 0.05, Figure 5B) and CD-DST assay (*p* < 0.05, Figure 5C). Both assays showed that DCTPP1 knockdown inhanced cisplatin sensitivity of ovarian cancer cells in both OVCAR8 and SKOV3 cells. Given that the main biological function of DCTPP1 is to maintain the safety of nucleic acids, comet assay was used to access the occurrence of double-strand DNA breaks (DSB), which is a natural progression of oxidative DNA damage lesions. Figure 5D shows that DCTPP1 knockdown lead to more long-tail cells in SKOV3 and OVCAR8 cells, which represent an increasing of DSB (*p* < 0.05). Then, we stained cells with Annexin V/PI following exposure to cisplatin with or without DCTPP1 knockdown, and Figure 5E shows that cisplatin-induced apoptosis was increased by DCTPP1 knockdown both in SKOV3 and OVCAR8 cells (*p* < 0.05). These results suggest that cisplatin-induced upregulation of DCTPP1 may be one of the mechanisms by which ovarian cancer resists the cytotoxicity of cisplatin. DCTPP1 knockdwon might enhence the cisplatin sensitivity of ovarian cancer cells and might be a promising therapeutic strategy.

## Discussion

DCTPP1, identified as a nucleoside triphosphate pyrophosphohydrolases, plays a critical role in the DNA repair mechanisms by eliminating harmful dCTP after DNA synthesis for the stabilization of dNTP pools and preventing oxidative damage of nDNA (16–20). In present study, we found protein expression level of DCTPP1 was significantly higher in OC tissues than in non-cancerous tissues and is associated with poor prognosis. More notably, patients with high expression of DCTPP1 showed a worse prognosis under cisplatin treatment. These results all show that DCTPP1 has the predictive potential for the ovarian cancer prognosis and chemotherapy efficacy.

In view of the powerful nucleic acid damage ability of ROS, cancer cells must prevent the accumulation of ROS to ensure the normal physiological metabolism (8–11). In this study, we verified that oxidative damage induced by cisplatin is the main factor of cytotoxicity, and then observed that cisplatin up-regulates the expression of DCTPP1 by generating ROS in ovarian cancer cells. These finding virified our speculation that the high expression of DCTPP1 may be in response to the intracellular ROS accumulation induced by cisplatin. These results give us confidence to further verify the role of DCTPP1 in cisplatin resistance.

First, we used lentivirus to deliver shRNA targeting DCTPP1 to construct the DCTPPI knockdown cell model of ovarian cancer cells. Through the cell models, we have observed that DCTPP1 is a promoter for the growth of ovarian cancer, which is consistent with the finding in prostate cancer and gastric cancer (Marquard and Jucker, 2020; Niu et al., 2021). With DCTPP1 knockdown, ovarian cancer cells showed growth inhibition both *in vivo* and *in vitro*. DCTPP1 inhibition also appears to have a negative impact on the survival and tumor malignancy of cancer cells. Our results show that DCTPP1 inhibition triggers a significant reduction in Akt activation (measured by the score of phosphorylated Akt/total Akt), and an ehancement in p21, p27 and PARP1 expression. As shown in previous studies, the activation of Akt signaling pathway and the inactivation of P21 are one of the reasons for the failure of chemotherapy, which further suggests the possibility that DCTPP1 might be involved in cisplatin resistance.

Under normal circumstances, the activation of PI3K/Akt pathway in tumor cells can trigger the increase of intracellular ROS and promote tumor malignant transformation, but ROS overload can reverse the activation of PI3K/Akt pathway through the expression of PTEN (Jaramillo and Zhang, 2013; Jang et al., 2016; Wu et al., 2020). Therefore, We verified whether the decrease in phosphorylated Akt levels observed during DCTPP1 inhibition due to some feedback mechanism in response to an increased ROS accumulation induced by shDCTPP1. While, the responses of the two ovarian cancer cells were inconsistent after knockdown of DCTPP1. OVCAR8 showed a significant increase in intracellular ROS, but SKOV3 showed no significant difference. Therefore, we cannot be sure that DCTPP1 knockdown can directly increase the level of intracellar ROS in ovarian cancer cells.

We further observed the relationship between DCTPP1 knockdown and intracellar redox homeostasis. DCTPP1 knockdown markedly decreased protein levels of Nrf2, GCLC, and HO-1 in both two cells. Nrf2 is a key redox regulator. HO-1 and GCLC are located downstream of Nrf2 and play important roles in intracellular redox balance as antioxidant enzymes (Bao et al., 2014; Silva et al., 2019; Deng et al., 2020; Ghosh et al., 2021). Some reports showed the balance between Nrf2/GSH antioxidant pathway and DNA repair might modulate cisplatin resistance in cancer cells (Qiu et al., 2020; Ghosh et al., 2021). The down-regulation of Nrf2 also corresponds to the decreased expression of its downstream targets HO-1 and GCLC, which suggest that DCTPP1 knockdown reduces cellular redox protection capacity through inhibiting the Nrf2 signaling pathway. Correspondingly, we have also observed a decline in the overall antioxidant capacity of cells in FRAP assay. At present, most reference believe that there is extensive interaction between the P13k/Akt signaling pathway and the Nrf2 pathway (Li et al., 2020; Qiu et al., 2020). From our results, it is not certain whether the reduction in Akt and Nrf2 activation induced by shDCTPP1 occur independently of each other, or whether one is upstream of the other. We tend to think that DCTPP1 may act as a redox-protected protein to help ovarian cancer cells respond to cisplatin-induced changes in intracellular ROS.

In the following experiments, we demonstrated that the sensitivity of ovarian cancer cells to cisplatin can be increased by inhibiting DCTPP1. As shown in our results, under the pressure of cisplatin, DCTPP1 knockdown significantly increased the accumulation of intracellular ROS. DCTPP1 knockdown increases the sensitivity of ovarian cancer cells to cisplatin in cisplatin-sensitive cells, which is reflected in growth inhibition, increased levels of apoptosis and increased DNA damage. Therefore, we speculate that cisplatin stimulates the overexpression of DCTPP1 in ovarian cancer cells to stabilize the accumulation of ROS in the cells, thereby protecting the cells from oxidative damage induced by cisplatin.

## Conclusion

High expression of DCTPP1 is associated with malignancy of ovarian cancer, and its expression could be induced by cisplatin. Under the pressure of cisplatin,up-regulation of DCTPP1 will minimize the oxidative DNA damage caused by ROS -overload. The clinical strategy against DCTPP1 may help to enhance the prognosis of cisplatin chemotherapy.

## Data Availability

The datasets presented in this study can be found in online repositories. The names of the repository/repositories and accession number(s) can be found in the article/Supplementary Material.

## Abbreviations

DCTPP1, dCTP pyrophosphatase 1; HO-1, heme oxygenase 1; GCLC, glutamate-cysteine ligase, catalytic subunit; Nrf2NF-E2, related factor 2; qPCR, quantitative PCR; RNAi, RNA interference; ROS, reactive oxygen species; shRNA, short harpin RNA; NTP-PPase, nucleotide triphosphate pyrophosphatase; NAC, N-acetyl-l-cysteine; CD-DST, 3D growth model build by collagen gel droplet culture technology.
